# Reactome enhanced pathway visualization

**DOI:** 10.1093/bioinformatics/btx441

**Published:** 2017-07-06

**Authors:** Konstantinos Sidiropoulos, Guilherme Viteri, Cristoffer Sevilla, Steve Jupe, Marissa Webber, Marija Orlic-Milacic, Bijay Jassal, Bruce May, Veronica Shamovsky, Corina Duenas, Karen Rothfels, Lisa Matthews, Heeyeon Song, Lincoln Stein, Robin Haw, Peter D’Eustachio, Peipei Ping, Henning Hermjakob, Antonio Fabregat

**Affiliations:** 1 European Molecular Biology Laboratory, European Bioinformatics Institute (EMBL-EBI), Wellcome Genome Campus, Hinxton, UK; 2 Ontario Institute for Cancer Research, Toronto, ON, Canada; 3 NYU Langone Medical Center, New York, NY, USA; 4 Department of Molecular Genetics, University of Toronto, Toronto, ON, Canada; 5 Department of Physiology, Medicine and Bioinformatics, NIH BD2K Center of Excellence, University of California, Los Angeles, CA, USA; 6 State Key Laboratory of Proteomics, Beijing Proteome Research Center, Beijing Institute of Radiation Medicine, National Center for Protein Sciences - Beijing, Beijing, China; 7 OpenTargets, Wellcome Genome Campus, Hinxton, UK

## Abstract

**Motivation:**

Reactome is a free, open-source, open-data, curated and peer-reviewed knowledge base of biomolecular pathways. Pathways are arranged in a hierarchical structure that largely corresponds to the GO biological process hierarchy, allowing the user to navigate from high level concepts like immune system to detailed pathway diagrams showing biomolecular events like membrane transport or phosphorylation. Here, we present new developments in the Reactome visualization system that facilitate navigation through the pathway hierarchy and enable efficient reuse of Reactome visualizations for users’ own research presentations and publications.

**Results:**

For the higher levels of the hierarchy, Reactome now provides scalable, interactive textbook-style diagrams in SVG format, which are also freely downloadable and editable. Repeated diagram elements like ‘mitochondrion’ or ‘receptor’ are available as a library of graphic elements. Detailed lower-level diagrams are now downloadable in editable PPTX format as sets of interconnected objects.

**Availability and implementation:**

http://reactome.org

## 1 Introduction

Pathway databases like Reactome systematically associate proteins with their functions and link them into networks that describe the reaction space of an organism. The basic unit of a pathway database is a reaction in which molecules are transformed. Transformations include the chemical changes of intermediary metabolism, as well as chemical modifications of proteins and other macromolecules, formation and reorganization of complexes, and transport events that move molecules from one cellular location to another. Pathways that accomplish more complex tasks like glycolysis or signal transduction mediated by a tyrosine kinase receptor, can be assembled from these reactions and can be grouped further to describe domains of biology like metabolism or signaling. Functional linkages between processes are consistently visible as shared molecules and dependencies, e.g. the output of one reaction is the input of another or positively regulates it, within or between pathways and domains.

Pathways in Reactome are organized hierarchically, grouping related detailed pathways (e.g. translation, protein folding and post-translational modification) into larger domains of biological function like metabolism of proteins. This hierarchical organization largely follows that of the Gene Ontology (GO) biological process hierarchy (Ashburner *et al.*, 2000; The Gene Ontology Consortium, 2015). Reactome pathways can be distinguished into two different types: higher level pathways (HLPs) that aggregate pathways within a similar biological process and detailed lower level pathways (LLPs) where the molecular processes are annotated as series of biomolecular reactions. Previously, HLP diagrams (HLDs) were simply implemented as a series of boxes symbolizing the subpathways, and allowing to navigate to them.

A formal data model such as the one embodied in Reactome makes pathways computationally accessible. Additionally, Reactome offers a pathway analysis service that supports enrichment and expression analysis (Fabregat *et al.*, 2016). Using the analysis service users can easily ask, e.g. whether the set of proteins up-regulated in a model system in response to a stress is distributed at random over reaction space or is clustered in particular domains, or could take advantage of the disease visualization to find out whether a mutation that blocks the function of a protein of interest would directly perturb any of the reactions that make up a process of interest. Additional tools are needed, however, to enable biologists to browse database content to visualize the relationships between parts of a domain or to explore possible relationships between domains.

Well-designed pathway visualization tools need to support diverse views providing different levels of detail. Dynamic navigation between views enables human users to visualize connections between pathways and domains (Suderman and Hallett, 2007). Diagrams can support inference more efficiently than equivalent linguistic representations (Perini, 2013). To facilitate interoperability it is also valuable for visualization tools to conform as much as possible to community standards like Systems Biology Graphical Notation (SBGN) (Le Novère *et al.*, 2009).

The challenge of pathway data visualization has been approached by several resources. In cases like MINERVA (Gawron *et al.*, 2016) and NAVICELL (Kuperstein *et al.*, 2013) the Google map engine was adopted to visualize pathways using SBGN. WikiPathways (Kutmon *et al.*, 2016) and KEGG (Kanehisa *et al.*, 2014) display pathways using an in-house developed viewer. Finally, other resources like Pathway Commons (Cerami *et al.*, 2011) display pathways as networks of gene–gene interactions. The most common navigation features used to explore pathway diagrams include zooming, panning and selection of pathway elements to view detailed information. Some tools, such as MINERVA or WikiPathways, also allow users to map drug targets or overlay experimental data. Another popular tool for pathway analysis and visualization is Ingenuity Pathway Analysis (https://www.qiagenbioinformatics.com), but as a commercial tool, it is inaccessible to many users.

**Fig. 1.**
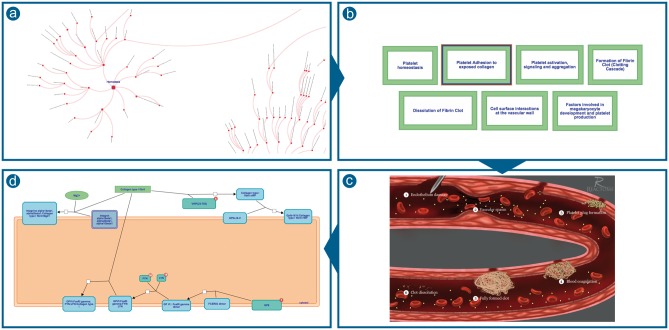
Reactome’s previous approach to scalable visualization **(a)** Pathways overview **(b)** HLDs were shown as a set of green boxes **(c)** Static images of biological processes illustrations were provided for some of the diagrams **(d)** SBGN-compliant diagrams containing molecular details of a reaction sequence

Reactome’s approach to scalable visualization environment, in place since 2015 (Fabregat *et al.*, 2016), provides multiple levels of detail as shown in Figure 1. A user starts with the ‘pathways overview’ view of all of reaction space, chooses to view the domain ‘haemostasis’, and within that domain chooses the pathway, ‘platelet adhesion to exposed collagen’. The first view is a graph of the entire Reactome event hierarchy (Fig. 1a); the final view shows the molecular details of a reaction sequence in a familiar SBGN-compliant format close to that of a classic metabolic map (Fig. 1d). The intermediate view, showing the pathways comprising haemostasis as a set of labeled green boxes (Fig. 1b), provides functionality for navigation and data analysis, but conceals the relevant biology, that this process is happening in a damaged blood vessel, that its parts occur in a causal sequence and that they involve complex interactions among molecules and cells in the blood and components of the vessel wall.

In addition to improved navigation, researchers have frequently requested options to export and save their pathways of interest in a format that can be reused for presentations, papers or other purposes, allowing them to easily manipulate the available pathway visualizations (e.g. alter the layout of a diagram or overlay the results of their research).

Here, we present the recent updates in the Reactome web interface providing improved visualization and navigation of Reactome pathways, as well as new options for downloading and re-using the pathway diagrams.

## 2 Implementation

**Fig. 2.**
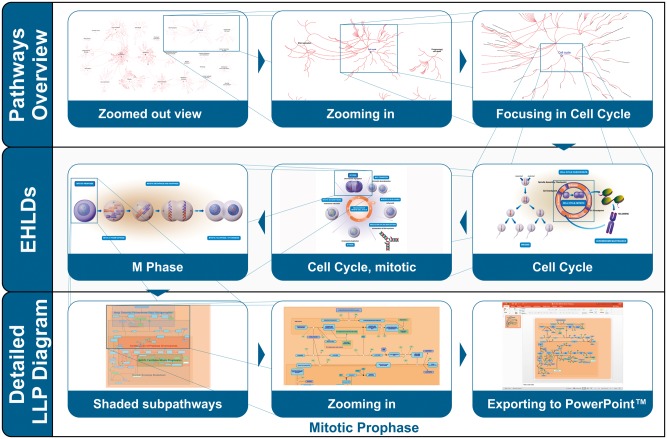
Different levels of visualization address different scenarios. The top row shows navigation from the entirety of reaction space to focus on Cell Cycle. The middle row presents EHLDs displaying successively narrower aspects of the cell cycle process. The bottom row presents navigation down to molecular details of individual reactions involved in mitotic prophase, and the optional export of this view to Microsoft PowerPoint

Aiming to address these challenges and boost the overall user experience, three new features were developed and integrated in the Reactome pathway diagram viewer (Fig. 2): (i) textbook-style enhanced high level diagrams (EHLDs), (ii) a mechanism to highlight different subpathways using coloured boxes in zoomed-out views of classic LLP diagrams and (iii) an option to export regular diagrams to PowerPoint.

Figure 2 presents the use of EHLDs to support a user who wants to navigate through the Cell Cycle domain of biology to arrive finally at the molecular details of pathway of interest, Mitotic Prophase. This top-down navigation of the Reactome pathways visualization begins with the Pathways Overview which provides a genome wide view of all the pathways in Reactome and their parent–child relationships. As the user progressively zooms in on Cell cycle, a series of EHLDs are displayed as interactive textbook-like illustrations that provide a visual representation of key biological concepts.

EHLDs aim to assist users to easily identify and focus on areas of interest by taking advantage of the human ability to easily perceive complex but visually obvious concepts. In this example, two levels of HLPs (Mitotic Cell Cycle and M Phase) are traversed through EHLDs, leading to the classic pathway diagram of the Mitotic prophase. This diagram, initially presented in its zoomed-out view, includes coloured boxes that highlight its different subpathways, helping the user to easily identify and comprehend the internal parts of this biological process. Subpathway highlighting progressively fades out as the user further zooms into the diagram and focuses on the reactions of a specific subpathway, for example Cisternae Pericentriolar Stack Reorganization.

### 2.1 Implementation of interactive EHLDs

Reactome previously used very simple sets of green box icons to facilitate navigation from highly abstract to more concrete, detailed levels of the pathway hierarchy (Fig. 1b). In some cases these were supplemented by static illustrations depicting the relationship of these pathways to each other (Fig. 1c). These static illustrations are often visually striking images of biological processes, but they lack interactivity; users cannot navigate to contained subpathways nor overlay summarized views of user data.

To enhance the HLP illustrations and make them interactive in the scope of the Reactome pathway browser, a number of technical and conceptual requirements had to be considered. Technically, the illustration had to be stored in a format that could be easily processed, while conceptually the illustration needed to provide a one-to-one mapping between the annotated pathway hierarchy and the graphical elements.

The task of producing these new EHLDs was assigned to a team of expert curators and illustrators who worked closely together to generate high quality overview diagrams for the higher levels of the Reactome pathway hierarchy. A common style and iconography was adopted wherever possible to enhance the ‘recognition effect’ and facilitate efficient navigation.

On the technical side, the SVG format was selected mainly due to the advantages it presents over other graphic formats such as PNG or JPEG. These advantages include (i) object-based vector representation for easy editing, (ii) resolution-independent zooming (Battiato *et al.*, 2005) suitable for the observation of large integrated pathways, (iii) interaction features for richer interfaces, (iv) data in regular text format as a subset of an eXtensible Markup Language (XML) that can be easily handled by computer programs or text editors and (v) support by most popular web browsers. Additionally, most popular graphic software packages used by designers, such as Adobe Illustrator, Corel Draw, Inkscape, can be used to import, edit and export in SVG format.

In the detailed LLP diagrams, the large number of entities and the variety of custom overlaid features made the usage of a multilayered HTML5 Canvas an appropriate technology (Miller *et al.*, 2013). However, since EHLDs have a limited number of entities, simple overlay features and a simpler rendering strategy, where the flow of information does not depend on the level of zoom, the adoption of an SVG renderer was deemed to be the best solution because modern browsers directly render this format. This allowed developers to focus on features such as overlays, zoom or translation which were implemented by applying a series of filters and transformations.

As part of the Reactome web interface, we have implemented an SVG rendering component that, beyond standard zooming and panning, allows specific regions of the diagram to be highlighted, recognizes mouse click events on specific regions to allow navigation to subpathways and allows analysis results to be overlaid onto the diagram. For the purposes of EHLDs, all of the required technical annotations were included in the SVG files by using the database id attribute of each relevant diagram element. In particular, three different types of technical annotations were used: (i) regions that represent a specific subpathway, (ii) labels containing the name of that subpathway and (iii) the region to be overlaid with the analysis results. Any element with no technical annotation was considered a decoration. The EHLD viewer reads the SVG file, renders the content and based on the technical annotations sets up the active regions, which can later be highlighted/selected or overlaid with analysis results. By interacting with any of the active regions representing subpathways, users can navigate to the respective subpathway diagram. The relevant code is part of the EHLD package available on the Reactome public GitHub repository (https://github.com/reactome-pwp/diagram). In addition, EHLDs are also available in the reusable stand-alone JavaScript diagram viewer (http://reactome.org/dev/diagram/js).

**Fig. 3.**
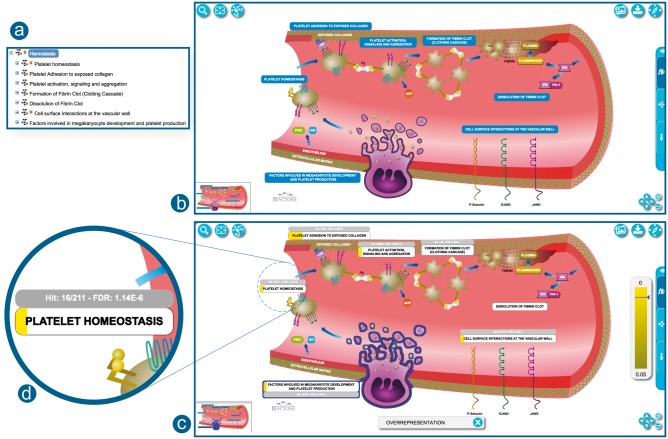
Correspondence between the hierarchy and EHLD. **(a)** Pathway hierarchy view for Haemostasis. **(b)** Haemostasis as an EHLD representation. **(c)** Haemostasis EHLD overlaid with pathway enrichment analysis results. The width of the yellow bar under the pathway label indicates the proportion of pathway entities contained in the analysed dataset. **(d)** A closer view to ‘Platelet Homeostasis’ label with analysis results overlaid. http://reactome.org/PathwayBrowser/#/R-HSA-109582

In the example EHLD presented in Figure 3b, the Haemostasis pathway hierarchy is no longer represented as a set of green boxes. Instead, the EHLD combines the illustration logic with a new design which includes a one-to-one mapping of its subpathways. This EHLD is processed by the software and placed as a fully interactive diagram skin, providing all the features of regular pathway diagrams such as hovering over elements, selection, flagging and pathway analysis results overlay. Moreover, the user interface provides fast zooming and panning support, with no image quality degradation. To provide easier in-diagram navigation, the viewer features a small thumbnail at the bottom-left corner of the viewport. EHLDs build on the power of illustrations to convey the depicted biological processes and their causal relationships, by adding interactivity and providing a rich user experience. For instance, the EHLD of Haemostasis (Fig. 3b) provides a clearer visual description regarding the role and order of each of the seven subpathways.

The viewer allows users to overlay pathway analysis results onto EHLDs (Fig. 3c). The results are displayed in the label of each subpathway. The label is overlaid by a coloured rectangular shape; its width and colour represent the percentage of hit entities and the *P*-value, respectively (Fig. 3d). Subpathways with a *P*-value below a certain threshold (*P* < 0.05) are coloured in grey. For hit subpathways, additional information about the hit elements and the false discovery rate are displayed next to the label. Upon hovering or selection of a subpathway, its *P*-value is indicated in the coloured legend bar displayed on the right side of the viewport. Analysis results are temporarily stored on the Reactome server. The storage period depends on usage of the service but is at least 7 days. Stored results are available via the token assigned to the results file when it is created and displayed in the URL for the results report. Users can easily share their view of Reactome with the results of their analysis overlaid by simply sharing the URL (Fabregat *et al.*, 2016).

**Fig. 4.**
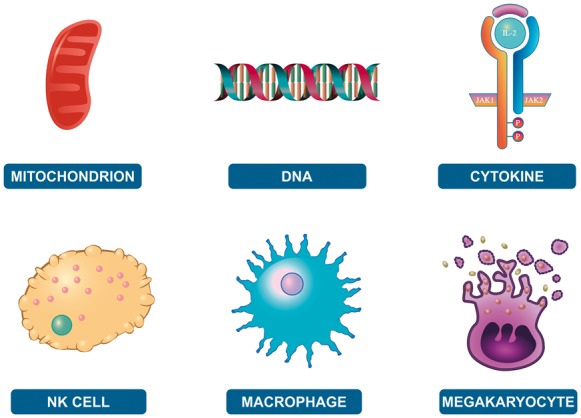
Example of different elements provided in the iconography library. http://reactome.org/icon-lib

EHLDs, including any analysis result overlay present at the time, can be easily downloaded and saved in SVG format. Users can edit the content of the exported files through the use of commercial or open-source graphics applications. EHLDs use a consistent iconography that reuses glyphs when the same entity plays a role in more than one biological process. For example, all platelets in the Haemostasis EHLD are represented by the same symbol. We have made a library of these graphical elements to provide them in SVG, PNG and EMF formats. The icon library is available at http://reactome.org/icon-lib and is distributed under the terms of the CC-BY license (https://creativecommons.org/licenses/by/4.0/). The aims of providing such a library are to facilitate the creation of uniform diagrams through the use of pre-existing glyphs and to offer these components to the community for reuse. Figure 4 presents a small sample of the elements that are available through the Reactome iconography library.

Researchers can use the provided icons to create their own illustrations to convey their findings and ideas, whether in a pathway diagram or a grant application illustration. Additionally, we would also like to encourage the community to contribute to the extension of this library through new glyphs (http://reactome.org/icon-info).

### 2.2 Subpathway highlighting

**Fig. 5.**
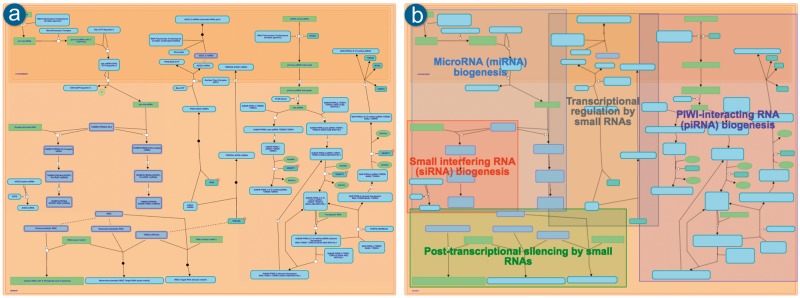
Pathway diagram for Gene silencing by RNA. **(a)** As it was displayed prior to the inclusion of the subpathways highlighting. **(b)** After adding the feature of highlighting the existing subpathways in the zoomed-out view (where the text in the entity icons is also omitted). http://reactome.org/PathwayBrowser/#/R-HSA-211000

Coloured boxes that highlight and distinguish subpathways within an LLD have been implemented to support easier navigation. Figure 5 compares the same pathway before (a) and after (b) the inclusion of this functionality. The boxes highlight the specific diagram reactions belonging to each subpathway, allowing further zooming in to the areas of interest within a given pathway diagram.

The position and colour of boxes is calculated on the server side at database release time and persists for the 3-month lifetime of the release. This strategy supports fast loading and ensures that the look and feel remains the same. The most complicated part of the task of automatic subpathway highlighting is deciding where to place the text inside each box. The adopted algorithm follows a basic space partitioning approach where the text is placed in the largest and widest possible rectangle inside each subpathway box after calculating the overlap with all the other subpathway boxes.

### 2.3 Export options

In addition to easy visualization and navigation, users often want to export/save pathways of interest in a format that can be reused for presentations, publications, or other purposes. In some cases, users want to use the pathway layout without modification, but in other cases, they may want to alter the diagram content to show the results of their own research or alter the layout. Exporting static images such as PNG files covers the first use case but to cover the second requirement, other formats are needed.

When exporting to other editable formats, there are several options to consider. A UX testing session with domain experts conceived the idea of exporting diagrams as an interconnected set of objects that adapt to layout changes. The main requirement was that when a glyph is moved around all the connected objects must follow e.g. when the user moves the glyph of a given reaction product, the reaction output line has to automatically readjust to keep pointing to it. Office tools such as Microsoft PowerPoint (http://office.microsoft.com/PowerPoint) are very common among biologists and allow the creation of multimedia presentations where the user can rearrange the contained objects and customize other properties such as size, colour or shape. By exporting Reactome diagrams to these tools in a standard format (PPTX), users can take advantage of all these features. Other options as SVG or PDF would export the content of a given diagram in a set of non-interconnected objects making changes in layout more difficult due to the need of manually moving every affected object.

Several issues were addressed to enable PPTX export. Reactome diagrams follow the SBGN standard, but the internal Reactome diagram data model does not define interconnections between different objects or anchor points between reactions, shapes and their participants. This absence of interconnection between elements needed to be programmatically addressed during the conversion phase to achieve movability of the contained objects without affecting their relationship with connecting lines. Different techniques were employed, including the use of invisible anchor points to join the segments of a reaction object and group the objects that visually define reaction properties (i.e. catalysis or regulation line endings).

**Fig. 6.**
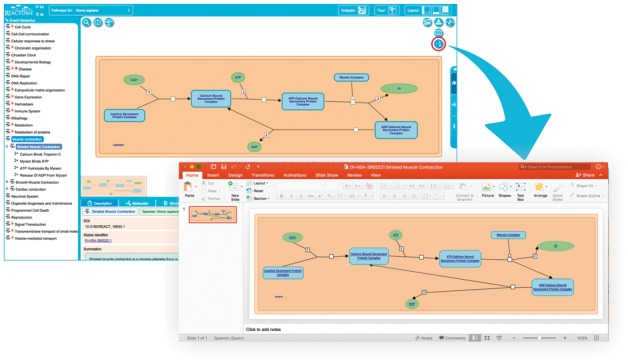
The pathway diagram for Striated Muscle Contraction exported to Microsoft PowerPoint. (http://reactome.org/PathwayBrowser/#/R-HSA-390522)

The PPTX files are generated on the server side, using the colour profile selected by the user on the client side. Storing the content in PPTX format is not straightforward, so Reactome utilized Aspose.Slides (https://www.aspose.com/products/slides/java), a commercial JAVA API for reading, writing and manipulating PowerPoint documents. Figure 6 presents an example of a regular diagram exported to Microsoft PowerPoint.

## 3 Discussion

The new features presented above have been included in the Reactome Pathway Diagram Viewer, which is fully integrated in the current version of the Reactome Pathway Portal. EHLDs and the option to export diagrams to PowerPoint are available to users accessing Reactome through the Pathway Portal or through the standalone version of the Diagram Viewer widget, which is available for integration in third party applications (http://reactome.org/dev/diagram).

Reactome curators and designers have created informative and visually appealing EHLD illustrations. Feedback from users regarding this new feature has been very positive. In particular, users found the new, interactive graphical representation of Reactome pathways more descriptive of the biological process and consequently more intuitive to navigate when compared to the previous static HLDs.

EHLDs have been designed to contain images that will be familiar to biologists. They use a consistent iconography that is based on a survey of typical textbook and online representations of the process. The intention is to make the navigation experience more intuitive and visually pleasing; the user will recognize the process that is represented and be able to select the appropriate region of the illustration to navigate to the next level of the Reactome hierarchy level without the need to read and understand text labels; ultimately the user will arrive at a classic, detailed pathway diagram that represents the molecular mechanism underlying the pathway. For users who are not familiar with the graphical representation used in EHLDs the text labels are retained.

Reactome users have responded positively to the inclusion of the subpathways highlight feature. Specifically, users found the new method of rendering LLDs (Fig. 5) more descriptive and less cluttered. The coloured boxes around subpathways give users a bird’s eye view of the displayed pathway, and to identify and later focus on regions of interest.

Apart from requesting enhancements of web visualization, users have often reported that the ability to export to PowerPoint would be a useful feature, mainly because it would enable them to conveniently open and edit Reactome pathway diagrams with the tool they use for creating posters and presentations. Feedback was particularly positive in relation to the ability to reposition diagram entities.

The limited visual appeal and navigability issues of previous Reactome HLDs have been addressed by the introduction of EHLDs, which represent biological processes in a familiar textbook style that allows intuitive navigation to more specific sub-topics. In addition, EHLDs are used to represent summarized analysis results. We have provided the ability to export EHLD images and associated analysis result overlays in a lossless, editable format, enabling users to represent their own research results in the context of Reactome pathway diagrams. In addition, the Reactome pathway iconography library provides graphical representations of common molecular biology elements suitable for use in slides and publications. Finally, classic pathway diagrams can now be exported in PPTX format, allowing their editing and reuse with familiar presentation software.

Regarding future work, in the short term Reactome curators and designers will replace all HLDs (ca. 85) with textbook-style EHLDs. In the mid-term, we plan to improve the integrated diagram search feature by taking advantage of Solr and our graph database (http://reactome.org/dev/graph-database). Finally, in a more generic fashion, we will continue improving the user interface to make it more user-friendly and responsive to the user’s behaviour and environment such as screen size, platform and orientation.

## Funding

National Institutes of Health BD2K grant (U54 GM114833); National Human Genome Research Institute at the National Institutes of Health (U41 HG003751); European Bioinformatics Institute (EMBL-EBI); Open Targets (The target validation platform); Medicine by Design (University of Toronto). Funding for open access charge: National Institutes of Health (U54 GM114833).

*Conflict of Interest*: none declared.
